# Molecular Mapping of Biofortification Traits in Bread Wheat (*Triticum aestivum* L.) Using a High-Density SNP Based Linkage Map

**DOI:** 10.3390/genes14010221

**Published:** 2023-01-14

**Authors:** Vasudha Jadon, Shashi Sharma, Hari Krishna, Gopalareddy Krishnappa, Rahul Gajghate, Narayana Bhat Devate, Kusuma Kumari Panda, Neelu Jain, Pradeep Kumar Singh, Gyanendra Pratap Singh

**Affiliations:** 1 Division of Genetics, ICAR-Indian Agricultural Research Institute, New Delhi 110012, India; 2 Amity Institute of Biotechnology, Amity University, Noida 201313, India; 3 ICAR-Sugarcane Breeding Institute, Coimbatore 641007, India; 4 ICAR-Indian Institute of Wheat and Barley Research, Karnal 132001, India; 5 National Bureau of Plant Genetic Resources, New Delhi 110012, India

**Keywords:** wheat, QTLs, SNPs, SSRs, candidate genes, mapping

## Abstract

A set of 188 recombinant inbred lines (RILs) derived from a cross between a high-yielding Indian bread wheat cultivar HD2932 and a synthetic hexaploid wheat (SHW) Synthetic 46 derived from tetraploid *Triticum turgidum* (AA, BB 2n = 28) and diploid *Triticum tauschii* (DD, 2n = 14) was used to identify novel genomic regions associated in the expression of grain iron concentration (GFeC), grain zinc concentration (GZnC), grain protein content (GPC) and thousand kernel weight (TKW). The RIL population was genotyped using SNPs from 35K Axiom^®^ Wheat Breeder’s Array and 34 SSRs and phenotyped in two environments. A total of nine QTLs including five for GPC (*QGpc.iari_1B*, *QGpc.iari_4A*, *QGpc.iari_4B*, *QGpc.iari_5D*, and *QGpc.iari_6B*), two for GFeC (*QGfec.iari_5B* and *QGfec.iari_6B*), and one each for GZnC (*QGznc.iari_7A*) and TKW (*QTkw.iari_4B*) were identified. A total of two stable and co-localized QTLs (*QGpc.iari_4B* and *QTkw.iari_4B*) were identified on the 4B chromosome between the flanking region of *Xgwm149–AX-94559916*. In silico analysis revealed that the key putative candidate genes such as P-loop containing nucleoside triphosphatehydrolase, Nodulin-like protein, NAC domain, Purine permease, Zinc-binding ribosomal protein, Cytochrome P450, Protein phosphatase 2A, Zinc finger CCCH-type, and Kinesin motor domain were located within the identified QTL regions and these putative genes are involved in the regulation of iron homeostasis, zinc transportation, Fe, Zn, and protein remobilization to the developing grain, regulation of grain size and shape, and increased nitrogen use efficiency. The identified novel QTLs, particularly stable and co-localized QTLs are useful for subsequent use in marker-assisted selection (MAS).

## 1. Introduction

Micronutrient and protein deficiency caused malnutrition is one of the important public health issues across the globe. Micronutrient deficiency (also referred to as hidden hunger) is a type of reduced or limited nutrition that results when the intake or absorption of minerals and vitamins is inadequate to support normal health and development in children and normal physical and mental function in adults. Globally, more than two billion people suffer from micronutrient deficiency alone [1]. World health organization recognized iron, zinc, and vitamin A as the three important limiting micronutrients in the global diet [2]. The primary cause of anemia or low hemoglobin content is iron deficiency, which affects nearly 40% of children under the age of 5 years and also 30% of pregnant women across the globe [3]. Anemia during gestation increases the risk of maternal death and low birth weight of the infants. Globally, each year around 2.5–3.4 million maternal and neonatal deaths were reported [4]. Iron is considered to be an important micronutrient for proper cognitive and motor development, further, young children and pregnant and lactating women are the most risk group for iron deficiency-related health issues.

Zinc is another important micronutrient, which stimulates the immune system and thereby increases the resistance against infectious diseases such as diarrhea, pneumonia, and malaria. Zinc is one of the important nutrients to support healthy gestation [5]. Approximately17.3% of the global population is at risk for zinc deficiency due to dietary insufficiency; around 30% of people are at risk in some countries or regions of the globe [6], causing the death of 0.09 million people and 9.1 million disability-adjusted life years in the year 2010 [7]. The nutritional and end-product quality of wheat is determined by both grain protein concentration and protein quality. One of the most common causes of various infections in humans is the decreased secondary immunity caused by protein energy malnutrition (PEM). Chronic PEM in children is clinically referred as marasmus (chronic wasting) or kwashiorkor (edema and anemia) [8]. Acute PEM leads to altered cognitive development in young children [9]. Micronutrient deficiency coupled with PEM are the major risk factors for losing health in developing nations, further, young children and pregnant women constitute the major risk groups [10]. In wheat, thousand kernel weight has no nutritional value per se; however, it does have a dilution effect on protein and micronutrient content. The difference in GPC can be partially attributed to the dilution effect due to increased grain yield [11]. Therefore, the improvement of TKW is always an important objective in wheat breeding programs due to its dual effects on both grain yield and grain quality.

Micronutrient malnutrition can be overcome through various interventions including dietary diversification, pharmaceutical supplementation, industrial fortification, and biofortification. The consumption of a diverse diet rich in micronutrients is one of the simplest and most effective strategies, however, affordability is an issue, especially for the economically weaker sections from developing and undeveloped countries. The other approaches including supplementation and fortification are not sustainable. Additionally, these interventions are linked with related difficulties including lack of fortified food availability to the desired and target people, and also lack of affordability, particularly economically weaker sections [12]. Hence, the approach of enhancing the nutrient status of food crops through crop breeding and transgenic interventions known as “genetic biofortification” emerged as a cost-effective and sustainable solution to alleviate micronutrient deficiencies. Effective policies to address micronutrient deficiencies including the application of micronutrient fertilizers and the development of nutri-rich crops through plant breeding are being considered as viable options [13]. Currently, the development of nutri-rich crop cultivars of staple crops is one of the top research priorities across the globe.

Wheat breeding programs must be re-orient to broaden the genetic base using landraces and crop wild relatives to effectively dissect the genetic basis of nutritional quality traits, and to develop wheat varieties with enhanced micronutrients and protein content [14]. Landraces are considered to be one of the important sources of wheat biofortification [15]. Higher grain zinc content has been successfully incorporated into elite breeding materials through a conventional breeding approach by the crossing of improved and adapted high-yielding wheat cultivars with *Aegilops tauschii*-derived SHWs or *Triticum spelta* accessions [16]. The iron and zinc status of modern cultivated wheat can be enhanced through the effective utilization of *Triticum dicoccoides* (wild emmer) in crop breeding programs [17]. *Triticum dicoccoides* derived *Gpc-B1* locus which was identified on the short arm of the 6B has a pleiotropic effect on grain protein, zinc, and iron content [18]. A NAC transcription factor (NAM-B1) encoded by an ancestral wild wheat locus *Gpc-B1* enhances nutrients including iron, zinc, and protein, probably by accelerating the senescence and thereby mobilization from leaves to developing grains [19]. Synthetic wheat developed from *Aegilops tauschii* has high grain zinc content and can serve as a valuable genetic resource to enhance grain zinc concentration in cultivated wheat [20].

Genetic dissection of complex traits such as GFeC, GZnC, GPC, and TKW is necessary to improve them through marker-assisted breeding (MAB). Detection of closely associated markers to quantitatively inherited traits would aid in the improvement of complex traits such as protein and micronutrients. Several studies have found a strong genotype-environment interaction in the expression of GFeC and GZnC [16,21], GPC and TKW [22,23]. Identification of genomic regions i.e., quantitative trait loci (QTLs) containing genes for grain protein, micronutrients, and TKW through molecular mapping in targeted mapping populations would allow plant breeders to develop biofortified varieties more efficiently.

The two most extensively utilized methods for determining the genetic basis of complex quantitative traits in agricultural crops are genome-wide association studies (GWAS) and quantitative trait loci (QTL) mapping. Extensive research efforts have been made in the last decade to discover QTLs linked with grain micronutrients, protein, and TKW through bi-parental based mapping populations in wheat [24,25,26,27,28,29,30,31,32,33]. The classical method of QTL mapping relies on structured populations like F_2_, RILs, back-crosses (BCs), and doubled haploids (DH). Similarly, GWAS has been successfully used to establish marker-trait association and identified the genomic regions associated with grain micronutrients, protein, and TKW [16,34,35,36,37,38,39,40,41,42,43,44] in wheat using a diverse set of genotypes in the GWAS panel. 

Although many mapping studies have been performed for yield and their component traits, only a few investigations on wheat nutritional quality traits have been undertaken. Furthermore, these traits are highly environment-sensitive, identification and validation of stable QTLs through multi-environment studies are of paramount importance to use them in MAB. Therefore, more systematic efforts may be necessary to identify the genetic mechanisms of nutritional quality traits in wheat and to devise marker-based breeding methods that involve the marker-assisted selection or genome-wide selection. The objective of the present study was to discover the novel genomic region(s) associated with GFeC, GZnC, GPC, and TKW using 188 RILs derived from HD2932 and synthetic 46.

## 2. Materials and Methods

### 2.1. Plant Material and Field Experiments

A set of 188 RILs derived from a cross between a high-yielding Indian bread wheat cultivar (HD2932: KAUZ/STAR//HD 2643) and a synthetic hexaploid wheat (Synthetic 46: Croc 1/*Ae. tauschii* (879)) derived from tetraploid *Triticum turgidum* (AA, BB 2n = 28) and *Triticum tauschii* (DD, 2n = 14) at CIMMYT, Mexico. The RILs in F_8_ and F_9_ were evaluated for GFeC, GZnC, GPC, and TKW. The RILs along with parental genotypes were tested at ICAR-Indian Agricultural Research Institute (IARI), New Delhi, India (28° 38′ N, 77° 9′ E, and 228.6 m AMSL) for two consecutive years during 2017–18 (F_8:9_), and 2018–19 (F_9:10_) in a randomized complete block design in two replications with three rows (1m length) per entry with a row-to-row spacing of 25 cm. The crop was planted under timely sown production conditions from 1–15th November during both years. Recommended package of practices were followed for raising the healthy crop with 150 kg of nitrogen (in the form of Urea and DAP), 60 kg of phosphorous (in the form of DAP), and 40 kg of potassium (in the form of Muriate of Potash) per hectare. As a basal dose, 50% N was applied at pre-planting and the remaining was applied in two split doses at 20–25 days and 40–45 days after sowing. Biotic stresses were optimally controlled with the application of efective fungicide (Tebuconazole 25% EC), pesticide (Imidacloprid 30.5 SC) and pre-emergence herbicide (Pendimethalin 30% EC).

### 2.2. Phenotyping for GFeC, GZnC, GPC, and TKW

After physiological maturity, a random sample of 25–30 spikes from each replicate was harvested manually. Approximately 20 g grains were sampled for micronutrient analysis and proper care was taken to avoid dust and metal contamination. A new cost-effective, non-disruptive, high throughput method called Energy Dispersive X-ray Fluorescence (ED-XRF) instrument (“Bench-top” X-Supreme 8000; Oxford Instruments plc, Abingdon, UK) available at ICAR-Indian Institute of Wheat and Barley Research (ICAR-IIWBR), Karnal, India was used for the estimation of GFeC and GZnC, which was expressed in milligrams per kilogram (mg/kg). The GPC was estimated by Infra-red transmittance-based instrument Infra-tec 1125 at (ICAR-IIWBR) and the values were expressed at 12% moisture basis. The Numigral grain counter was used to count the grains and the weight of the 1000 grains was measured in weighing balance.

### 2.3. Genotyping

Genotyping data and linkage map were obtained from the available map [45] with the following details. The parental genotypes and RILs genomic DNA were extracted from 20–25 day old seedlings using CTAB method [46]. Hybridization-based 35 K SNP chip makers from Axiom wheat breeders’ array and simple sequence repeat (SSR) markers were used for genotyping. SNP detection from 35 K Axiom^®^ Wheat Breeder’s Array of Affymetrix GeneTitan^®^ system was carried out according to the procedure described by Affymetrix. Allele calling was carried out using Affymetrix proprietary software package Axiom Analysis Suite, following the Axiom^®^ Best Practices Genotyping Workflow (https://media.affymetrix.com/support/downloads/manuals/axiom_analysis_suite_user_guide.pdf, accessed on 3 March 2022). SSR markers of *Xcfd*, *Xcfa*, *Xgwm*, *Xgdm*, *Xbarc*, and *Xwmc* series were used as described by Gajghate (2021) [45].

The polymerization chain reaction was carried out in a total volume of 20 µL, with the components including 1× PCR buffer (100 mM Tris-HCl with pH 8.8; 500 mM KCl; 1% Triton X-100; 16 mM MgCl_2_), template DNA (10 ng), dNTP mix (0.02 mM), forward and reverse primer (5 pM each), Taq polymerase (0.3-unit, Bangalore genie, Bengaluru, India).The amplified PCR products were resolved in 3.5% agarose or 4% metaphor agarose gel (under low-resolution conditions) at 120 V for 3 h in TBE buffer. For the construction of a framework linkage map, polymorphic SSR and SNP markers between the parents were binned and finally a set of 836 high-quality markers including 802 SNPs and 34 SSRs were placed in linkage groups by the program IciMapping v 4.2.53 software [47]. Kosambi mapping function was used to convert recombination frequencies in cM values [48]. The final map was drawn using the online program MG2C v.2.1 [49].

### 2.4. Statistical Analysis and QTL Mapping

Descriptive statistics and analysis of variance (ANOVA) were calculated with Microsoft Excel and agricolae package in R (https://www.r-project.org/, accessed on 18 June 2022). The ggplot2, corrplot and basic R program were used to generate frequency distribution curves, box plots, and person’s correlation plots. QTL mapping was done following Inclusive composite interval mapping (ICIM) using IciMapping v 4.2.53 software (http://www.isbreeding.net, accessed on 8 October 2022). Environment-wise and pooled phenotypic data of each genotype were used along with a linkage map for QTL identification. Missing phenotypic data were set to deletion in ICIM and the walking speed was 1.0 cM, with *p* = 0.001 in step-wise regression. A manual LOD threshold at 2.5 was used to detect QTLs. Flanking markers of QTLs with their respective position in cM, along with threshold LOD and PVE were obtained. The standard procedure was followed to name the QTLs [50].

### 2.5. In Silico Analysis

The sequence information of the significant SNPs and SSRs flanking QTLs were utilized to search for putative candidate genes with Basic Local Alignment Search Tool (BLAST) using default parameters in the ensemble plants platform (http://plants.ensembl.org/Triticum_aestivum/Tools/Blast, accessed on 28 October 2022) of the bread wheat genome (Wheat Chinese Spring IWGSC RefSeq v1.0 genome assembly (2018)). The genes found in the overlapping and the region of 0.5 Mb downstream of the left marker and upstream of the right markers were identified as putative candidate genes. The role of the identified genes in the regulation of grain micronutrients, GPC, and TKW was also determined through earlier studies.

## 3. Results

### 3.1. Variability and Correlations

**Figure 1:**
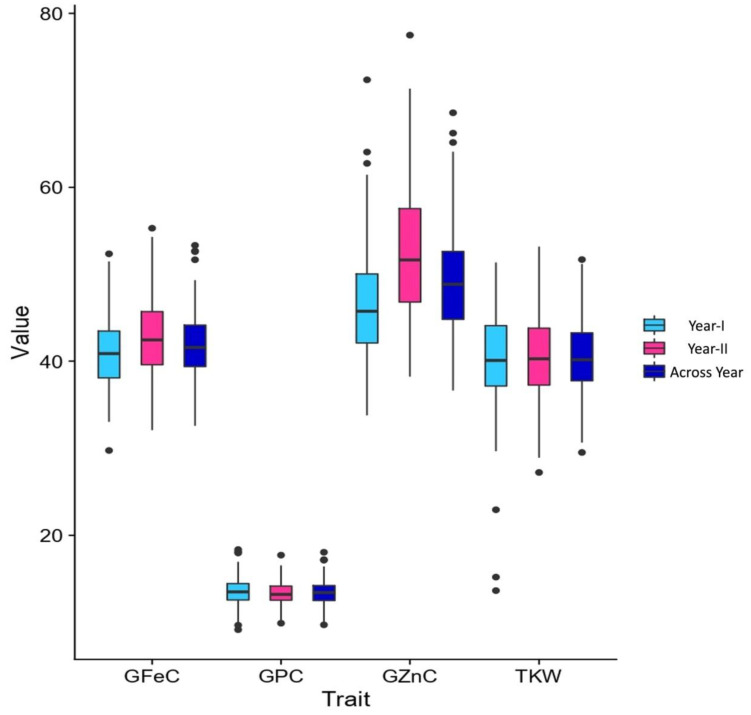
Boxplots for GFeC, GZnC, GPC, and TKW in RIL population grown at ICAR -IARI during 2017–18 (year I) and 2018–2019 (year II) and across years. GFeC: grain iron concentration in (mg/kg); GZnC: grain zinc concentration in (mg/kg); GPC: grain protein content (%); TKW: thousand kernel weight.

**Figure 2:**
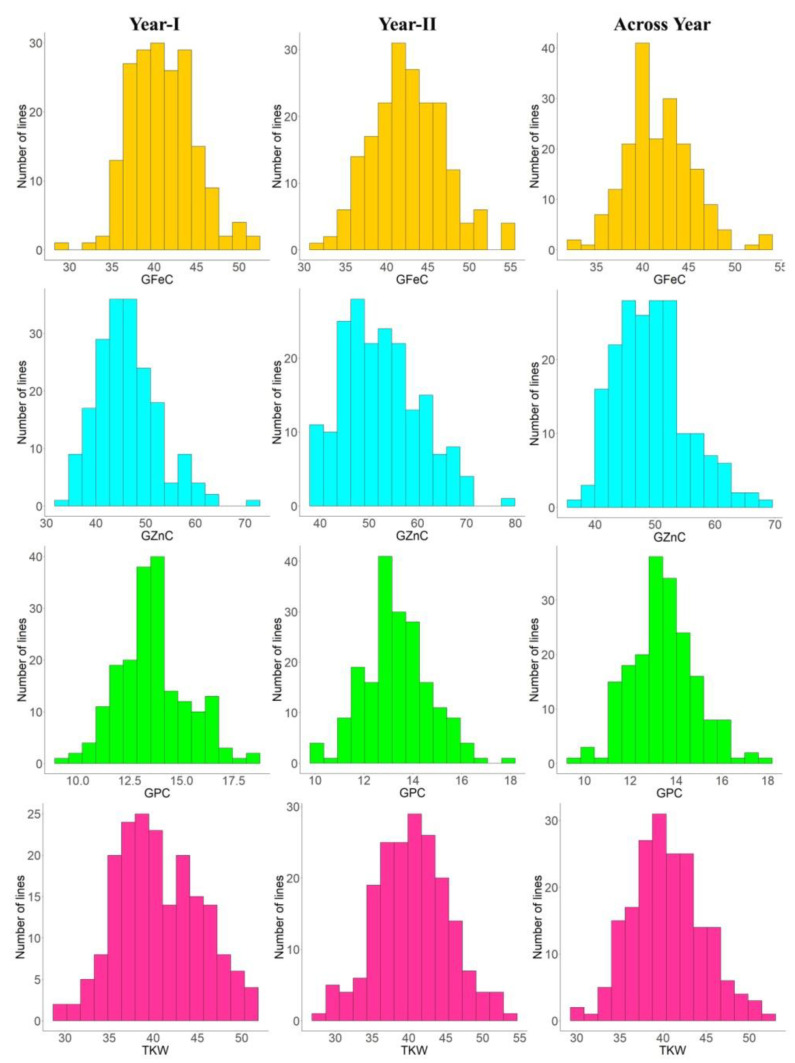
Frequency distributions for GFeC, GZnC, GPC, and TKW in the RIL population grown at ICAR-IARI during 2017–18 (year I) and 2018–2019 (year II) and across years.

**Figure 3:**
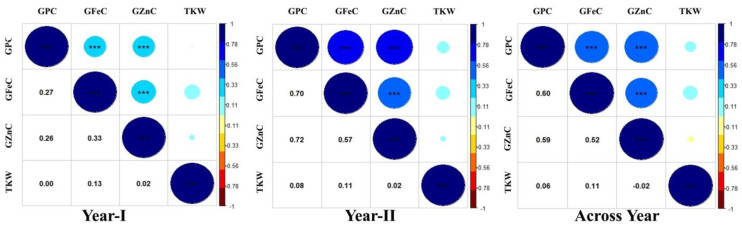
Genetic correlation coefficients among GFeC, GZnC, GPC, and TKW in RIL population grown at ICAR-IARI during 2017–18 (year I) and 2018–2019 (year II) and across years. *** significant values at *p* < 0.001.

The heritability and variance parameters of the RIL population along with parents are presented in Table 1. The parental genotype i.e., Synthetic46 had high trait values for all the traits in both the tested environments compared to the other parental genotype (HD2932). A wide range of variation has been observed for all the traits in both the environments for GFeC, GZnC, GPC, and TKW ranging from 29.75–55.30 mg/kg, 33.80–77.50 mg/kg, 09.16–18.38%, and 25.20–53.17 gm, respectively. The percent coefficient of variation was higher during year II compared to year I for all the traits except TKW. Superior performing RILs along with parents were given in supplementary Table S1. Similarly, trait-wise highest CV was recorded for GZnC, followed by TKW, GFeC and GPC, and exactly the reverse trend was observed for all the traits with respect to broad sense heritability. The genetic advance was also highest for GZnC, followed by TKW, GFeC, and GPC. The graphical presentation of the mean is given as a box plots in the Figure 1. Transgressive segregants were observed for all the studied traits in both directions (Figure 1). The environment or year effect was more pronounced for grain micronutrients compared to TKW and GPC. The frequency distribution of grain micronutrients, TKW, and GPC in the RIL population tested in year I and year II are presented in Figure 2. The RILs population exhibited continuous and near-normal distribution for all the studied traits. Pearson’s correlation coefficient (r^2^) of grain micronutrients, TKW, and GPC was determined and presented in Figure 3. The correlation among GFeC, GznC, and GPC was found to be highly significant and positive in both the tested environments and across environments, however, the correlation of TKW with the other three traits is neutral.

### 3.2. Genome-Wide Marker Distribution

**Figure 4:**
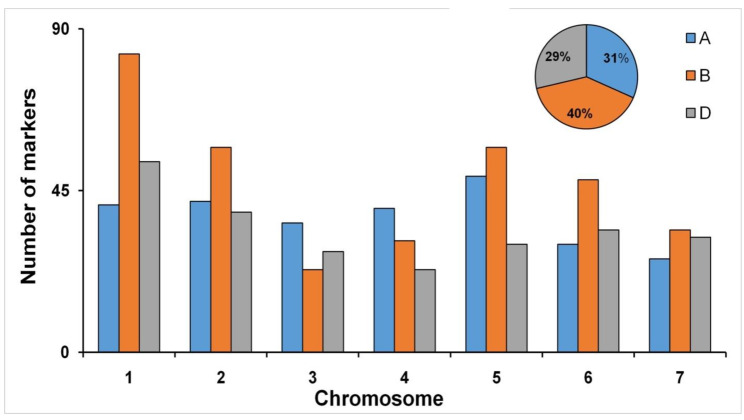
Histogram for the number of polymorphic markers distributed across chromosomes; pie-chart represents the percentage of marker distributed on A, B, and D genome. Blue colour indicates A genome; Orange colour indicates B genome; Grey colour indicates D genome.

High-quality SNPs was obtained by processing the 35K SNP array. As a result, a total of 802 high quality genome-wide SNPs along with 34 SSRs were further utilized for QTL mapping analysis. The chromosome and subgenome -wide distribution of the genetic marker are presented in Figure 4. The highest number of genetic markers were mapped on subgenomeB (333), followed by A (264) and D (239) subgenomes. Chromosome-wise distribution of markers ranged from 23 (3B and 4D) to 83 (1B) within the subgenome.

### 3.3. Quantitative Trait Locus (QTL) Mapping

**Figure 5:**
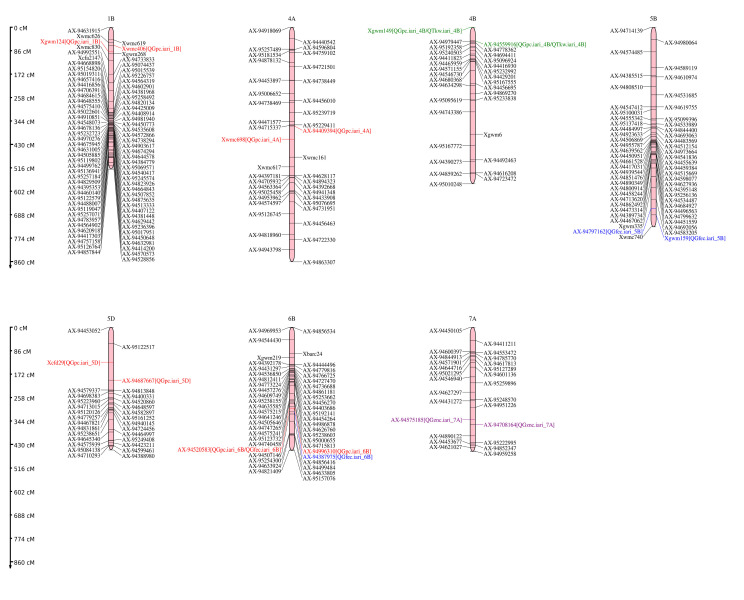
QTL positions identified in A, B, and D subgenomes of RILs derived from HD2932 × synthetic 46. Blue colour indicates QTLs for GFeC; purple colour indicates QTLs for GZnC; red colour indicates QTLs for grain GPC; green colour indicates QTLs for TKW.

A set of nine QTLs were identified for GFeC, GZnC, GPC, and TKW for year I, year II and across years. The identified QTLs were mapped on 1B, 4A, 4B, 5B, 5D, 6B, and 7A chromosomes. The details of the identified QTLs are presented in Table 2 and illustrated QTL positions in the linkage map in Figure 5. The highest number of QTLs were identified for GPC (5 QTLs) which were located on 1B, 4A, 4B, 5D, and 6B followed by GFeC (2 QTLs) which were mapped on 5B and 6B. Similarly, one QTL was identified for GZnC on 7A, and one QTL for TKW on 4B chromosomes. Also, a genomic region flanking between *Xgwm149–AX-94559916* harbours co-localized QTLs for both GPC and TKW. 

#### 3.3.1. QTL Mapping for Grain Micronutrients

Two QTLs associated with the expression of GFeC were identified on chromosomes 5B and 6B, whereas, one QTL associated with the expression of GZnC was identified on the 7A chromosome. *QGfec.iari_5B* flanked between *AX-94797162–Xgwm159* identified in the year I and across years were mapped at a confidence interval of 670.5–698.5 cm on 7B chromosome with the explained phenotypic variation of 9.0 and 6.7%. The second QTL associated with GFeC (*QGfec.iari_6B*) was explained 5.2% phenotypic variation, which was flanked between *AX-94520583–AX-94387975* at the confidence interval of 292.5–305.5 cm. Also, one QTL (*QGznc.iari_7A*) associated with GZnC was identified on 7A and flanked between *AX-94575185–AX-94708164* at a confidence interval of338.5–363.5 cm with the explained phenotypic variation of 6.6%. All the identified QTLs had positive alleles from the Synthetic 46 parent except *QGfec.iari_6B*, which had alleles from the parentHD2932.

#### 3.3.2. QTL Mapping for GPC and TKW

The highest number of 5 QTLs associated with the expression of GPC were identified in the year I, II and across years. Two QTLs i.e., *QGpc.iari_1B* and *QGpc.iari_4A* identified in year I along with across years were mapped between *Xwmc406–Xgwm124* and *AX-94409394–Xwmc698* at a confidence interval of 60.5–84.5 cm and 371.5–409.5 cm, respectively. These two QTLs explained phenotypic variations of 4.9 and 10.0%. Similarly, two QTLs i.e., *QGpc.iari_5D* and *QGpc.iari_6B* were identified in one environment (year II) and mapped between the flanking markers of *Xcfd29–AX-94687667* and *AX-94996310–AX-94520583* at a confidence interval of 128.5–159.5 cm and 293.5–302.5 cm, respectively. These two QTLs explained the phenotypic variation of 10.7 and 5.6%. One QTL (*QGpc.iari_4B*) was identified on 4B with the explained phenotypic variation ranging from 3.7–7.4%. This QTL was identified at a confidence interval of 0–21.5 cm on 4B between the flanking region of *Xgwm149–AX-94559916.* One QTL **(***QTkw.iari_4B*) was identified for TKW at a confidence interval of 0–12.5 cm between the flanking region of *Xgwm149–AX-94559916.* The identified QTL explained the phenotypic variation of 10.5% and 13.4%. All the identified QTLs had positive alleles from the Synthetic 46 parent except *QGpc.iari_5D* and *QGpc.iari_6B* which had alleles from the HD2932.

#### 3.3.3. Stable and Co-Localised QTLs 

A total of two stable QTLs for GPC and TKW were identified in the present study. One QTL i.e., *QGpc.iari_4B* was identified in both the tested environments (year I and year II) along with pooled mean at a confidence interval of 0–21.5 cm. The other stable QTL (*QTkw.iari_4B*) was identified for TKW on 4B at a confidence interval of 0–12.5 cm. Also, both of these stable QTLs were co-localized between the flanking region of *Xgwm149–AX-94559916* on 4B chromosome.

### 3.4. Identification of Putative Candidate Genes

Table 3 shows the SNP and SSR markers linked with grain micronutrients, GPC, and TKW that were used to identify the putative candidate genes using the annotated wheat reference sequence (RefSeq v1.0). The functional role of some of the important putative candidate genes was also discussed. One QTL i.e., *QGfec.iari_6B* associated with GFeC encode L-aspartate oxidase (TraesCS6B02G127300) and F-box domain (TraesCS6B02G086000). Similarly, one QTL i.e., *QGznc.iari_7A* associated with GZnC encode P-loop containing nucleoside triphosphate hydrolase (TraesCS7A02G041000), Protein kinase domain (TraesCS7A02G000900), Nodulin-like protein (TraesCS7A02G000800), NAC domain (TraesCS7A02G000300). Three QTLs i.e., *QGpc.iari_1B*, *QGpc.iari_4A*, and *QGpc.iari_6B* associated with GPC encode Purine permease (TraesCS1B02G413500), Zinc-binding ribosomal protein (TraesCS4A02G019000), Cytochrome P450 (TraesCS4A02G019400), Protein phosphatase 2A (TraesCS4A02G341600), GDSL lipase/esterase (TraesCS4A02G341500), Zinc finger, CCCH-type (TraesCS6B02G167200). The grain protein QTL *QGpc.iari_4B* encodes Kinesin motor domain (TraesCS4B02G269800).

## 4. Discussion

Although several QTLs/MTAs have been detected for yield and associated traits, only a few QTLs were identified for nutritional and end-product quality traits in wheat. Therefore, more systematic attempts may be necessary to uncover the genetic basis of nutritional quality traits and to devise marker-aided breeding methods involving MAS. Furthermore, quality traits are highly environment-sensitive and identification of stable QTLs through multi-environment studies is of paramount importance to use them in varietal improvement programmes through MAS. Also, the wheat genome is highly complex, and there is always the possibility to detect novel genomic regions for quality traits.

Significant effects of environment and genotype-environment interactions (GEI) were observed in the expression of GFeC, GZnC, GPC, and TKW. GZnC was the most environment-sensitive trait, whereas GPC was relatively a stable trait with minimum environmental influence. High intensity of environmental and GEI effects have also been reported in earlier studies for the expression of GFeC and GZnC [21,60,61], GPC and TKW [22,23,62]. The intensity of environmental and GEI effects is an important factor in the detection of environment-specific as well as consistent QTL(s). The significant and positive correlation among GFeC, GZnC, and GPC observed in the present study were also reported in earlier studies [35,63]. However, the correlation of TKW with the other three traits is neutral. Therefore, all three associated traits (GFeC, GZnC, and GPC) can simultaneously be improved in the breeding programmes. Through conventional breeding approach, high-grain zinc content has already been successfully transferred to elite breeding material from *Aegilops tauschii*-based synthetic hexaploid wheats (SHWs) or *Triticum spelta* accessions. Previously, *Triticum dicoccoides* derived *Gpc-B1* locus on chromosome 6B has been found to have a pleiotropic effect on GFeC, GZnC, and GPC [18]. In the present study also, two QTLs (*QGfec.iari_6B* and *QGpc.iari_6B*) were identified on the 6B chromosome for GFeC and GPC. Coincidentally, one of the parents used in the development of RIL populations in the present study is a synthetic hexaploid wheat.

The framework map was developed with 836 high-quality informative markers including 802 SNPs and 34 SSRs in a set of 188 RILs spanning a map length of 10,913 cm and utilized for the QTL analysis [45]. A total of 9 QTLs including 5 for GPC (*QGpc.iari_1B*, *QGpc.iari_4A*, *QGpc.iari_4B*, *QGpc.iari_5D*, *QGpc.iari_6B*), 2 for GFeC (*QGfec.iari_5B* and *QGfec.iari_6B*), and one each for GZnC (*QGznc.iari_7A*) and TKW (*QTkw.iari_4B*) were identified. The highest number of QTLs were identified in subgenome B (6 QTLs), followed by subgenome A (2 QTLs) and subgenome D (1 QTL). The subgenome-wise distribution of QTLs is similar to the marker distribution pattern of subgenomes, as the maximum markers were mapped on subgenome B (333) followed by subgenome A (264), and the lowest number of markers (239) were mapped in subgenome D. 

The identified QTLs for GFeC were mapped on 5B and 6Bchromosomes between the flanking regions of *AX-94797162–Xgwm159* and *AX-94520583–AX-94387975,* respectively. The association of genomic regions for GFeC on chromosome 5B was also reported in the previous study by Liu et al. (2019) [27]. The other grain micronutrient (GZnC) was identified on 7A chromosome between the flanking region of *AX-94575185–AX-94708164*, the same chromosome harbours the zinc QTLs in the earlier studies [27,28,29,39]. The highest number of 5 QTLs were identified for GPC, the identified QTLs were mapped on 1B, 4A, 4B, 5D, and 6B chromosomes. The association of genomic regions for GPC on chromosomes 1B [64,65,66,67], 4A [27,65,66,68], 4B [64,67,68,69,70], 5D [64,68,69], and 6B [70] was also reported in previous studies. Similarly, the QTL identified on the 4B chromosome for TKW was also reported in the same chromosome at different positions and different marker interval in the earlier studies also [65,68]. All the identified 9 QTLs in the present study are novel, as the earlier reported QTLs were mapped at different locations and different marker intervals. The genomic region flanked between *Xgwm149–AX-94559916* could be a potential candidate region, as it harbours two stable and co-localized QTLs (*QGpc.iari_4B* and *QTkw.iari_4B*). 

The various putative candidate genes underlying QTLs for grain micronutrients, GPC, and TKW were detected through BLAST search (Table 3). The QTLs detected in different chromosomes were located in gene-coding regions related to zinc finger, transcription factors, transmembrane proteins, and kinase-like superfamilies. For example, *QGznc.iari_7A* associated with GZnC encodes P-loop containing nucleoside triphosphatehydrolase (TraesCS7A02G041000) found to have a role in zinc ion binding. Similarly, a Nodulin-like protein (TraesCS7A02G000800) was found to have a role in iron homeostasis in arabidopsis [51] and zinc transportation in maize [52]. Another important putative candidate gene i.e., NAC domain (TraesCS7A02G000300) found to have a definite role in Zn, Fe, and protein remobilization to the developing grain [19], translocation of iron, zinc, and nitrogen from vegetative tissues to grain [53], Zn and Fe remobilization to seeds in rice [54]. Putative candidate genes underlying QTLs for GPC were also identified, *QGpc.iari_1B* encodes Purine permease (TraesCS1B02G413500), which is found to have a role in regulating grain size via modulating cytokinin transport in rice [55]. Another QTL (*QGpc.iari_4A*) encodes Zinc-binding ribosomal protein (TraesCS4A02G019000), Cytochrome P450 (TraesCS4A02G019400), and Protein phosphatase 2A (TraesCS4A02G341600) have a role in the binding of barley grain proteins [56], regulates grain size by affecting the extent of integument cell proliferation [57], increased nitrogen use efficiency in Rice [58], respectively. Similarly, *QGpc.iari_6B* encodes Zinc finger CCCH-type (TraesCS6B02G167200) found to have a role in the regulation of GluB-1 promoter and controls the accumulation of glutelins protein during grain development in rice [59]. Also, *QGpc.iari_4B* flanked between *Xgwm149-AX-94559916* encodes Kinesin motor domain (TraesCS4B02G269800) found to have a role in the regulation of grain shape in rice [60].

Similarly, some of the putative candidate genes associated with the GFeC, GZnC, GPC, and TKW, identified in the present study also reported in previous reports. For instance, putative candidate gene i.e., P-loop containing nucleoside triphosphate hydrolase associated with grain zinc concentration was reported [25,63]. Similarly, putative candidate gene NAC domain associated with metal and nutrient remobilisation in grains was identified [54,71]. Another putative candidate gene i.e., Cytochrome 450 associated with high grain protein content in wheat lines derived from wild emmer wheat was identified [72]. The putative candidate genes including Zinc finger, CCCH-type and Cytochrome 450 are also associated with quality traits in wheat including grain iron, protein, gluten content, baking value, hardness index and sedimentation value [35,73].

## 5. Conclusions

The study with 188 RILs revealed that GFeC, GZnC, GPC, and TKW were quantitatively inherited traits. The strong positive correlation among grain micronutrients and GPC suggested the possibility of improving these traits simultaneously. A set of nine QTLs including five for GPC, two for GFeC, and one each for GZnC and TKW were identified. Also, a total of two stable and co-localized QTLs were identified in more than one environment and associated with the expression of GPC and TKW. Several putative candidate genes encoding important functions such as iron homeostasis, zinc transportation, Zn, Fe, and protein remobilization, regulating grain size regulation of grain size and shape, and increased nitrogen use efficiency. Further validation and functional characterization of the candidate genes to elucidate the role of these genes in wheat is envisaged. 

## Figures and Tables

**Table 1:** Mean, heritability, and variance parameters of GFeC, GZnC, GPC, and TKW.

Trait	Env.	HD2932	SYN46	RIL Population						
				Range	Mean + S.D	CV%	h^2^ (bs)	GCV	ECV	GA
GFeC	Year-I	35.10	45.05	29.75–52.35	40.96 ± 1.85	04.52	48.46	08.64	04.53	6.46
	Year-II	32.40	46.25	32.10–55.30	42.61 ± 3.63	08.53	50.23	08.57	08.53	5.33
	Across years	33.75	45.65	32.60–53.32	41.79 ± 2.76	06.62	55.23	07.35	06.62	4.70
GZnC	Year-I	46.95	56.30	33.80–72.35	46.30 ± 4.00	08.63	65.98	12.02	08.63	9.31
	Year-II	43.21	58.45	38.25–77.50	52.55 ± 6.67	12.69	47.96	12.18	12.69	9.13
	Across years	45.08	57.37	36.65–68.57	49.42 ± 5.32	10.76	44.57	09.65	10.76	6.56
GPC	Year-I	11.61	13.35	09.16–18.38	13.50 ± 0.59	04.32	88.26	11.84	04.32	3.11
	Year-II	10.16	13.60	09.90–17.73	13.33 ± 0.86	06.48	65.52	08.93	06.48	1.98
	Across years	10.88	13.47	09.71–18.05	13.45 ± 0.75	05.60	75.00	07.35	06.62	4.70
TKW	Year-I	34.93	46.15	25.20–51.35	40.36 ± 6.55	16.22	51.26	15.97	16.22	2.35
	Year-II	35.43	46.27	27.24–53.17	40.48 ± 3.40	08.39	61.18	10.54	08.39	6.87
	Across years	35.18	46.21	26.22–52.26	40.54 ± 3.39	08.36	49.85	08.34	08.36	4.91

Year-I: 2017–18; Year-II: 2018–19; CV: coefficient of variation; h^2^ (bs): heritability (broad sense); GCV: genotypic coefficient of variation; PCV: phenotypic coefficient of variation; GA: genetic advance.

**Table 2:** List of QTLs identified for GFeC, GZnC, GPC, and TKW.

Trait	QTL Name	Env.	Position	Flanking Markers	LOD	PVE (%)	Add	Confidence Interval
GFeC	*QGfec.iari_5B*	Year I	680	*AX-94797162–Xgwm159*	3.9	09.0	1.42	670.5–698.5
		Across Years	681	*AX-94797162–Xgwm159*	2.7	06.7	1.10	670.5–686.5
	*QGfec.iari_6B*	Across Years	299	*AX-94520583–AX-94387975*	2.9	05.2	−0.97	292.5–305.5
GZnC	*QGznc.iari_7A*	Year I	349	*AX-94575185–AX-94708164*	2.8	06.6	1.98	338.5–363.5
GPC	*QGpc.iari_1B*	Year I	72	*Xwmc406–Xgwm124*	2.6	04.9	0.50	60.5–84.5
		Across Years	72	*Xwmc406–Xgwm124*	2.6	04.9	0.50	60.5–84.5
	*QGpc.iari_4A*	Year I	389	*AX-94409394–Xwmc698*	2.7	10.0	0.72	371.5–409.5
		Across Years	389	*AX-94409394–Xwmc698*	2.7	10.0	0.72	371.5–409.5
	*QGpc.iari_4B*	Year I	0	*Xgwm149–AX-94559916*	3.0	03.7	0.44	0–21.5
		Year II	0	*Xgwm149–AX-94559916*	5.0	07.4	0.42	0–14.5
		Across Years	0	*Xgwm149–AX-94559916*	3.0	03.7	0.44	0–21.5
	*QGpc.iari_5D*	Year II	141	*Xcfd29–AX-94687667*	2.7	10.7	−0.50	128.5–159.5
	*QGpc.iari_6B*	Year II	298	*AX-94996310–AX-94520583*	3.9	05.6	−0.37	293.5–302.5
TKW	*QTkw.iari_4B*	Year I	0	*Xgwm149–AX-94559916*	3.7	10.5	1.77	0–11.5
		Year II	0	*Xgwm149–AX-94559916*	5.6	13.4	1.75	0–12.5

GFeC: grain iron concentration; GZnC: grain zinc concentration; GPC: grain protein content; TKW: thousand kernel weight; Year I: 2017–18; Year II: 2018–19; Positive value indicates that the allele was inherited from Synthetic 46, and negative value indicates that the allele was inherited from HD2932.

**Table 3:** List of putative candidate genes identified for GFeC, GZnC, GPC and TKW.

Trait	QTL Name	Marker Interval	TraesID	Putative Candidate Genes	Functions
GFeC	*QGfec.iari_6B*	*AX-94520583-AX-94387975*	TraesCS6B02G127300	L-aspartate oxidase	–
			TraesCS6B02G086000	F-box domain	–
GZnC	*QGznc.iari_7A*	*AX-94575185–AX-94708164*	TraesCS7A02G041000	P-loop containing nucleoside triphosphate hydrolase	Zinc ion binding
			TraesCS7A02G000900	Protein kinase domain	–
			TraesCS7A02G000800	Nodulin-like protein	Iron homeostasis in arabidopsis [51], Zinc transportation in Maize [52]
			TraesCS7A02G000300	NAC domain	Zn, Fe and Protein remobilization to the developing grain [19]. Translocation of iron, zinc, and nitrogen from vegetative tissues to grain [53], Zn and Fe remobilization to seeds in Rice [54]
GPC	*QGpc.iari_1B*	*Xwmc406–Xgwm124*	TraesCS1B02G413500	Purine permease	Regulates grain size via modulating cytokinin transport in rice [55]
	*QGpc.iari_4A*	*AX-94409394–Xwmc698*	TraesCS4A02G019000	Zinc-binding ribosomal protein	Binding of barley grain proteins [56]
			TraesCS4A02G019400	Cytochrome P450	Regulates grain size by affecting the extent of integument cell proliferation [57]
			TraesCS4A02G341600	Protein phosphatase 2A	Increased nitrogen use efficiency in Rice [58]
			TraesCS4A02G341500	GDSL lipase/esterase	–
	*QGpc.iari_6B*	*AX-94996310–AX-94520583*	TraesCS6B02G167200	Zinc finger, CCCH-type	Regulation of GluB-1 promoter and controls the accumulation of glutelins protein during grain development in Rice [59]
TKW & GPC	*QGpc.iari_4B*	*Xgwm149-AX-94559916*	TraesCS4B02G269800	Kinesin motor domain	Grain shape in rice [60]

## Data Availability

Data is contained within the article.

## Supplementary Materials

The following supporting information can be downloaded at: https://www.mdpi.com/article/10.3390/genes14010221/s1, Table S1: superior recombinant lines along with parental values of all the studied traits.
