# Supplementary material for: Molecular Mapping of Biofortification Traits in Bread Wheat (Triticum aestivum L.) Using a High-Density SNP Based Linkage Map
Source: Genes (Basel). 2023 Jan 14;14(1):221. doi: 10.3390/genes14010221 (PMC9859277; doi:10.3390/genes14010221)
Supplement: Supplementary file 1 [file genes-14-00221-s001.zip › genes-2155794-supplementary.pdf]

Trait	Environment	Parents		10 Superior RILs									
GPC		P1	P2	RIL-68	RIL-54	RIL-124	RIL-30	RIL-164	RIL-166	RIL-36	RIL-47	RIL-181	RIL-65
	2017-18	13.15	11.62	18.38	17.97	18.13	16.55	16.14	16.90	16.38	16.18	16.34	16.14
	2018-19	14.35	10.69	17.73	16.46	16.15	16.29	16.55	15.75	15.66	15.75	15.58	15.75
	Pooled	13.75	11.15	18.05	17.21	17.14	16.42	16.34	16.33	16.02	15.97	15.96	15.94
GFeC		P1	P2	RIL-164	RIL-30	RIL-119	RIL-65	RIL-24	RIL-27	RIL-54	RIL-174	RIL-175	RIL-179
	2017-18	45.05	35.10	52.35	51.48	49.90	49.25	49.85	45.45	48.10	47.00	47.95	46.20
	2018-19	43.90	35.95	54.30	53.90	55.30	54.10	48.80	51.60	48.25	49.25	47.70	48.85
	Pooled	44.48	35.53	53.33	52.69	52.60	51.68	49.33	48.53	48.18	48.13	47.83	47.53
GZnC		P1	P2	RIL-68	RIL-112	RIL-181	RIL-24	RIL-47	RIL-21	RIL-65	RIL-22	RIL-166	RIL-178
	2017-18	56.30	46.95	59.65	64.05	60.25	62.75	56.75	72.35	59.30	61.45	58.00	53.64
	2018-19	57.00	47.95	77.50	68.40	70.05	65.45	69.70	52.85	65.55	63.15	66.45	69.70
	Pooled	56.65	47.45	68.58	66.23	65.15	64.10	63.23	62.60	62.43	62.30	62.23	61.67
TKW		P1	P2	RIL-30	RIL-20	RIL-64	RIL-14	RIL-47	RIL-176	RIL-75	RIL-100	RIL-84	RIL-24
	2017-18	37.80	39.10	50.20	51.35	49.75	51.20	51.20	49.20	45.75	47.58	48.13	50.08
	2018-19	42.13	41.20	53.17	51.05	52.01	48.94	47.96	48.21	51.41	49.06	48.24	46.28
	Pooled	39.97	40.15	51.69	51.20	50.88	50.07	49.58	48.71	48.58	48.32	48.18	48.18

S1: superior recombinant lines along with parental values of all the studied traits
